# Comparisons of high-risk cervical HPV infections in Caribbean and US populations

**DOI:** 10.1186/1750-9378-4-S1-S9

**Published:** 2009-02-10

**Authors:** Camille C Ragin, Angela Watt, Nina Markovic, Clareann H Bunker, Robert P Edwards, Stacy Eckstein, Horace Fletcher, David Garwood, Susanne M Gollin, Maria Jackson, Alan L Patrick, M Smikle, Emanuela Taioli, Victor W Wheeler, Jacque B Wilson, N Younger, Norma McFarlane-Anderson

**Affiliations:** 1 Department of Epidemiology, University of Pittsburgh Graduate School of Public Health, Pittsburgh, USA; 2 Division of Cancer Prevention and Population Science, University of Pittsburgh Cancer Institute, Pittsburgh, USA; 3 Faculty of Medical Sciences, University of the West Indies, Kingston, Jamaica; 4 Division of Gynecologic Oncology, University of Pittsburgh Cancer Institute, Pittsburgh, USA; 5 Department of Human Genetics, University of Pittsburgh Graduate School of Public Health and University of Pittsburgh Cancer Institute, Pittsburgh, USA; 6 Tobago Branch, Trinidad and Tobago Cancer Society, Scarborough, Tobago, Trinidad & Tobago; 7 Tobago Health Studies Office, Scarborough, Tobago, Trinidad & Tobago; 8 Department of Epidemiology and Biostatistics, Downstate School of Public Health, State University of New York, USA

## Abstract

**Background:**

Disparities in cervical cancer incidence and mortality rates exist among women of African ancestry (African-American, African-Caribbean and African). Persistent cervical infection with Human papillomavirus (HPV) is associated with cervical dysplasia and if untreated, could potentially progress to invasive cervical cancer. Very few studies have been conducted to examine the true prevalence of HPV infection in this population. Comparisons of cervical HPV infection and the type-specific distribution of HPV were performed between cancer-free Caribbean and US women.

**Results:**

The Caribbean population consisted of 212 women from Tobago and 99 women from Jamaica. The US population tested, consisted of 82 women from Pittsburgh. The majority of the US subjects was Caucasian, 74% (61/82) while 12% (10/82) and 13% (11/82) were African-American or other ethnic groups, respectively. The age-adjusted prevalence of any HPV infection among women from Tobago was 35%, while for Jamaica, it was 81% (p < 0.0001). The age-adjusted prevalence of HPV infection for Caribbean subjects was not statistically significantly different from the US (any HPV: 47% vs. 39%, p > 0.1; high-risk HPVs: 27% vs. 25%, p > 0.1); no difference was observed between US-Blacks and Jamaicans (any HPV: 92% vs. 81%, p > 0.1; high-risk HPV: 50% vs. 53%, p > 0.1). However, US-Whites had a lower age-adjusted prevalence of HPV infections compared to Jamaican subjects (any HPV: 29% vs. 81%, p < 0.0001; high-risk HPV: 20% vs. 53%, p < 0.001). Subjects from Jamaica, Tobago, and US-Blacks had a higher proportion of high-risk HPV infections (Tobago: 20%, Jamaica: 58%, US-Blacks: 40%) compared to US-Whites (15%). Similar observations were made for the presence of infections with multiple high-risk HPV types (Tobago: 12%, Jamaica: 43%, US-Blacks: 30%, US-Whites: 8%). Although we observed similar prevalence of HPV16 infections among Caribbean and US-White women, there was a distinct distribution of high-risk HPV types when comparisons were made between the ethnic groups.

**Conclusion:**

The higher prevalence of cervical HPV infections and multiple high-risk infections in Caribbean and US-Black women may contribute to the high incidence and prevalence of cervical cancer in these populations. Evaluation of a larger sample size is currently ongoing to confirm the distinct distribution of HPV types between ethnic groups.

## Background

Disparities in cervical cancer incidence and mortality rates exist among all women of African ancestry (African-American, African-Caribbean and African) [1,2]. The world standardized age-adjusted incidence and mortality rates per 100,000 for African-American women are 8.6 and 3.4, respectively vs. 6.7 and 1.7 for Caucasians in the United States (US) [1]. Furthermore, African and Caribbean countries have the top two highest incidence and mortality rates, above Asia, the Americas and Europe [2].

Almost all cases of invasive cancers of the cervix, most other anogenital tract cancers [3], and approximately 36% of oropharyngeal cancers [4], are associated with HPV infection. In addition to persistent HPV infection with high-risk genotypes, access to screening as well as behaviors such as early age of sexual intercourse, multiple sex partners, and lifetime number of partners are risk factors associated with the development of cervical cancer. Although the HPV infection rate is high in the Caribbean [5], very few studies have been conducted to measure the true prevalence of HPV infection in this population. In addition, there are not enough data that describe the distribution of HPV types among cancer cases and healthy controls from the many islands throughout the Caribbean. Since the HPV vaccine specifically prevents cancers caused by HPV16 and 18, knowledge of the HPV type distribution in these populations is important in order to evaluate the impact of the current HPV vaccine on these at-risk populations.

We have previously reported the HPV type distribution in two cancer-free Caribbean populations, Jamaica [6] and Tobago [7]. In this study, we have compared the prevalence and genotype distribution of cervical HPV infections in these two Caribbean populations with that of US women.

## Methods

### Study population

The Caribbean populations consisted of 212 women from Tobago [6] and 99 women from Jamaica [7]. The US population consisted of 88 women from Pittsburgh. All of the subjects from the three geographic areas were recruited from the general population and none were pregnant. The women from Tobago were recruited between July and September 2004 by means of posters, flyers, public service announcements on television and radio, word of mouth, and a series of cancer information sessions conducted throughout the island. The Jamaican women were recruited during visits to a family practice in Western Jamaica between January 2003 and October 2006. All Jamaican participants were recruited consecutively to avoid selection bias. The US study population was enrolled from May 2007–Aug 2008 as part of a nested cross-sectional study of the Epidemiologic STudy of HEalth Risk (ESTHER) project, (an ongoing women's study at the University of Pittsburgh). The ESTHER project's sample recruitment methodology attempted to address some of the biases related to use of a sample of convenience. The targeted recruitment for ESTHER was based on age, level of education, and race/ethnicity population distributions. Recruitment strategies included attending women focused events, news items and advertisements in women-focused news sources, and limited respondent driven sampling.

All subjects from Jamaica and Tobago combined are referred to in this study as Caribbean. The majority of subjects were African-Caribbean, although in Tobago, there were 19 subjects who defined their ethnicity as East Indian. The self-reported ethnic groups in the US population were classified as White, Black or other ethnic groups. One subject did not define her ethnicity and was included in the other ethnic group category.

### HPV testing

Standardized protocols were implemented for sample collection, DNA extraction and HPV testing for all three sub-populations that were included in this analysis. A nurse or clinician collected cervical brush samples and the DNA was extracted from these samples using the Puregene DNA purification kit (Qiagen, Germantown, MD, USA). The HPV genotyping was performed on these samples using the Linear Array HPV Genotyping kit (Roche Diagnostics). All cervical samples were tested at the University of Pittsburgh. The assay involved amplification of samples by PCR using a master mix which contained biotin-labeled primers for the detection of the 37 most common HPV genotypes as well as the human beta-globin gene. The PCR products were chemically denatured and hybridized for 30 min at 53°C to linear array strips which contained specific and one cross-reactive oligonucleotide probe for the HPV genotypes as well as a high and low concentration of a beta-globin probe. The HPV genotypes were identified when visualized using a streptavidin-horseradish peroxidase conjugate and a substrate solution containing hydrogen peroxide and 3,3',5,5'-tetramethylbenzidine which yielded a blue precipitate at the positions where the hybridization occurred.

For six samples from the US population, beta-globin was not positive, which indicated either poor quality or low yield DNA samples. These samples were excluded. Therefore, HPV results were available for only 82 US samples. All of the samples from Jamaica were tested with the AMPLICOR HPV method [7] prior to testing with the Linear Array HPV Genotyping kit. Ten samples from the Jamaican population tested HPV-negative using our assay, possibly due to a lower sensitivity. Therefore, for our study, these samples were classified as HPV X.

The Linear Array protocol does not specifically detect HPV52. The cross-reactive probe in this assay detects HPV 33, 35, 52 and 58 combined. According to the manufacturer's protocol, samples that are negative for HPV 33, 35, and 58 individually, but positive for the cross-reactive probe are classified as HPV 52-positive. Samples that are positive for HPV 33, 35, and/or 58 individually, as well as the cross-reactive probe have an uncertain HPV 52 status. For our study these samples were considered negative for HPV 52.

HPV-risk classification was based on the epidemiological classification of HPV types that are associated with cervical cancer [8]. Briefly there are fifteen high-risk HPV types: 16, 18, 31, 33, 35, 39, 45, 51, 52, 56, 58, 59, 68, 73, 82; three probable high-risk HPV types: 26, 53, 66 and twelve low-risk HPV types: 6, 11, 40, 42, 43, 44, 54, 61, 70, 72, 81, CP6108. For this analysis, we combined probable high-risk HPV types and high-risk HPV types into a single group. The HPV types that do not fall into any of these aforementioned categories were classified as undetermined risk.

### Statistical analysis

All statistical analyses were performed using STATA SE (version 10), (StataCorp LP, College Station, TX). Age-adjusted prevalence rates and confidence intervals were calculated using logistic regression estimates of infection with any HPV type or any high-risk HPV type, adjusted for age. Observations with missing values of age were dropped from the analysis before estimation. The chi-squared test of proportions was used to calculate p-values for the unadjusted differences in proportions as well as, p-values for the differences in adjusted proportions. Although age and age at first sexual intercourse were both normally distributed, we rejected the hypothesis of equal variances. Therefore, the comparisons of mean age and age at first sexual intercourse were performed using two-sample t-tests on the equality of means adjusting for unequal variances.

## Results

### Study populations

Table 1 summarizes the characteristics of each subpopulation. The overall study population consisted of 393 subjects (mean age 42 ± 12.7 years) who were recruited from the general population. The Caribbean subjects consisted of 212 women from Tobago [6] (mean age: 41 ± 11.6 years). The 99 women from Jamaica [7] (mean age: 36 ± 11.7 years) were the youngest population, while the US population, which consisted of 82 women (mean age: 53 ± 9.0 years) was the oldest (p < 0.0001). The majority of subjects in the US population were White, 76% (67/88), while 11% (10/88) and 13% (11/88) were African-American or other ethnic groups, respectively.

**Table 1:** Characteristics of the study population.

	**Tobago**	**Jamaica**	**US**	**Total**
Number of subjects	212	99	82	393
Population source	GP	GP	GP	GP
Age (years, mean ± SD)	41 ± 11.6	36 ± 11.7 (n = 91)	53 ± 9.0	42 ± 12.7 (n = 391)
Age, first sexual intercourse (years, mean ± SD)	18 ± 3.5 (n = 191)	17 ± 2.8 (n = 95)	19 ± 3.8 (n = 80)	18 ± 3.5 (n = 366)
Number of sex partners N (%)				
1–5	153 (79.7)	72 (74.2)	48 (60.8)	273 (74.2)
6–15	39 (20.3)	20 (20.6)	24 (30.4)	83 (22.6)
16+	0 (0.0)	5 (5.2)	7 (8.9)	12 (3.3)

GP = general population; SD = standard deviation; when there are subjects with missing data, the number of the subpopulation (n) used to calculate the overall means is indicated in each cell.

The mean age at first sexual intercourse for each subpopulation ranged from 17 to 19 years old, with an overall mean of 18 ± 3.5 years. The age at first sexual intercourse for Jamaican women was statistically significantly younger than women from Tobago (Jamaica: 17 ± 2.8 years vs. Tobago: 18 ± 3.5 years, p = 0.01) and US women (vs. US: 19 ± 3.8 years, p = 0.0001).

### Comparisons of cervical HPV prevalence between countries

Thirty-five percent (75/212) of the women from Tobago, 84% (83/99) of the women from Jamaica and 32% (26/82) of US women, tested positive for HPV infection of any HPV type. Table 2 summarizes the age-adjusted prevalence of HPV infections in our study population. Although the age-adjusted prevalence rate of any HPV infection was higher overall in the Caribbean (47%), it was not significantly different from the United States (39%, p = 0.281). Similar observations were made when only high-risk HPV infections were considered (Caribbean: 27%, US 25%, p = 0.758). The highest prevalence of high-risk HPV infections was observed in Jamaican women (age-adjusted prevalence = 53%), while women from Tobago had a statistically significantly lower age-adjusted prevalence of high-risk HPV infections (18%, p < 0.0001). Statistically significant differences were observed between the age-adjusted prevalence of high-risk HPV infections among US Whites (20%) and Jamaican women (p = 0.001). In contrast, there was no statistically significant difference in the prevalence of high-risk infections among US-Blacks (50%) when compared to Jamaican subjects (p > 0.1).

**Table 2:** Prevalence of HPV infection by geographic location.

Country	Test method	Age-adjusted prevalence any HPV (%), 95% CI	p-value, Country	p-value, Ethnic group	Age-Adjusted prevalence high-risk HPV (%), 95% CI	p-value, Country	p-value, Ethnic group
Caribbean	PCR	47.1 (41.3–53.0)	ref		27.4 (22.3–33.3)	ref	
Jamaica		80.5 (70.5–87.8)		ref	52.7 (41.4–63.6)		ref
Tobago		34.5 (28.3–41.2)		<0.0001	18.3 (13.5–24.2)		<0.0001
United States	PCR	39.4 (28.2–51.8)	0.281		25.3 (15.5–38.4)	0.758	
US-Blacks		91.8 (58.3–98.9)		0.368	49.7 (21.4–78.2)		0.867
US-Whites		29.0 (18.0–43.0)		<0.0001	20.2 (10.9–34.7)		0.001
US-Other		22.9 (5.9–58.5)		0.002	16.0 (2.3–61.0)		0.111

p-values for the differences in adjusted proportions.

### Geographical distribution of high-risk HPV types and multiple high-risk HPV infections

**Figure 1:**
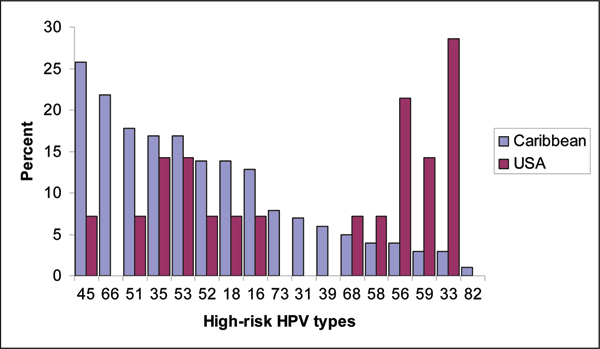
Distribution of high-risk HPV types among Caribbean and US women with high-risk HPV infections.

Figure 1 summarizes the HPV type distribution of high-risk HPV infections among all women infected with high-risk HPV types. In both Caribbean islands, HPV 45 (overall: 26%, Tobago: 19% and Jamaica: 31%) rather than HPV 16 (overall: 13%, Tobago: 12% and Jamaica 14%) or HPV 18 (overall: 14%, Tobago: 5% and Jamaica: 21%) was the most common high-risk genotype detected. In contrast, US women had a different distribution of high-risk genotypes. HPV 33 infections were highest among the high-risk types in this population (overall: 29%, US-Blacks: 25%, US-Whites: 22%) rather than HPV 16 (overall: 7%, US-Blacks: 0%, US-Whites: 11%) or HPV 18 (overall: 7%, US-Blacks: 0%, US-Whites: 11%). Nevertheless, although not statistically significant, we observed a higher prevalence rate of HPV 16 and 18 infections among Caribbean subjects that were infected with high-risk HPV types when compared to the US women (HPV 16: 13% vs. 7%, p > 0.1 and HPV 18: 14% vs. 7%, p > 0.1).

The proportion of high-risk HPV infections appears to be highest among women of African ancestry. Jamaican women had the highest proportion of high-risk HPV infections (58.6%), then US-Black (40%), Tobago (20.3%) and US White (14.5%) women, followed by women from the US-other group (10%), (Table 3). Fifty percent of US-black women, 40.4% of Jamaican women, 23.1% of Tobago women, 16.1% of US-white (40.4%) and 10% of women from the US-other group had single high-risk HPV infections. Similar observations were made for multiple high-risk HPV infections, US-whites and US-other had lower proportions (8.1% and 10%, respectively). Higher proportions of multiple infections were observed among Jamaicans (43.4%), US-blacks (30%) and women from Tobago (12.3%). In general, multiple high risk infections among Caribbean women ranged from 2–6 high-risk HPV types whereas no more than two high-risk HPV types were detected among US Blacks.

**Table 3:** Characteristics of HPV infections according to ethnicity.

	Tobago N N (%)	Jamaica N (%)	US-Black N (%)	US-White N (%)	US-other N (%)	Total N (%)
Number of subjects	212	99	10	67	11	393
High-risk HPV infection	43 (20.3)	58 (58.6)	4 (40.0)	9 (14.5)	1 (10.0)	115 (29.3)
Low-risk HPV infection	15 (7.1)	25 (25.3)	4 (40.0)	4 (6.5)	1 (10.0)	49 (12.5)
Undetermined-risk infection	16 (7.6)	47 (47.5)	5 (50.0)	5 (8.1)	1 (10.0)	74 (18.3)
Single high-risk infection*	49 (23.1)	40 (40.4)	5 (50.0)	10 (16.1)	1 (10.0)	105 (26.7)
Multiple high-risk infection	26 (12.3)	43 (43.4)	3 (30.0)	5 (8.1)	1 (10.0)	78 (19.9)
Number of high-risk HPV types per subject						
None**	169 (79.7)	41 (41.4)	6 (60.0)	53 (85.5)	9 (90.0)	278 (70.7)
1	33 (15.6)	34 (34.3)	3 (30.0)	7 (11.3)	1 (10.0)	78 (19.9)
2	7 (3.3)	11 (11.1)	1 (10.0)	2 (3.2)	0 (0.0)	21 (5.3)
3	2 (0.9)	7 (7.1)	0 (0.0)	0 (0.0)	0 (0.0)	9 (2.3)
4	1 (0.5)	2 (2.0)	0 (0.0)	0 (0.0)	0 (0.0)	3 (0.8)
5	0 (0.0)	3 (3.0)	0 (0.0)	0 (0.0)	0 (0.0)	3 (0.8)
6	0 (0.0)	1 (1.0)	0 (0.0)	0 (0.0)	0 (0.0)	1 (0.3)

*Includes subjects infected with low-risk and undetermined-risk HPV types; **Includes subjects that were HPV-negative or had only low-risk HPV infections.

## Discussion

This is the first study to report comparisons of the prevalence of cervical HPV infections and the specific HPV genotypes between cancer-free Caribbean and US women. We have shown that the age-adjusted prevalence rate of any HPV infection was higher in the Caribbean women (47%) than in women from the United States (39%), although the age-adjusted prevalence of high-risk HPV infections (Caribbean 27%, US 25%) was similar in both populations. The inability to achieve statistical significance for high-risk HPV infections may be related to the small sample size of the US population and consequently insufficient statistical power to detect a difference between the two groups. Nevertheless, our findings suggest that the higher rates of HPV infection in the Caribbean compared to the US, are likely to explain the higher incidence of cervical cancer reported in the region [2].

The distribution of HPV types was similar in the two Caribbean islands but was different from that observed in the US. This is not unexpected, since differences in the geographical distribution of HPV infections have previously been reported [9]. We observed that HPV 16 and 18 were not the predominant high-risk genotypes detected in cancer-free Caribbean women. Our results show that Caribbean women have a higher proportion of HPV 45 infections, the third most common high-risk HPV genotype that drives cervical carcinogenesis [8,10]. Studies are currently ongoing to determine whether HPV 45 infections might be driving cervical cancer development in the Caribbean region.

The current HPV vaccine prevents infections from HPV types 6, 11, 16 and 18 [11]. The low-risk genotypes, HPV 6 and 11, are responsible for the development of genital warts, while HPV 16 and 18 are the two most common high-risk genotypes and are reported to account for almost 70% of cervical cancers worldwide [10]. We observed that HPV 16 and 18 infections are not seen in high proportions among cancer-free Caribbean women. However, Caribbean women still have a higher proportion of HPV 16 and 18 infections compared to their US counterparts (HPV 16: 13% vs. 7%, and HPV 18: 14% vs. 7%). These observed differences between the US and Caribbean may be due to immune resistance to HPV16 and 18 genotypes in the US population (although we do not have data to support these hypotheses). The evaluation of serological status for HPV 16 and 18 and how these compare to the prevalence of high-risk HPV infections in both geographical regions are needed in order to test these hypotheses. Nevertheless, the higher incidence of cervical cancers in the Caribbean region compared to the US might be attributed to the higher proportions of HPV 16 and 18 infections observed in the Caribbean.

Caribbean and US-Black subjects in comparison with US-Whites have higher prevalence rates of high-risk HPV infections and also a higher proportion of subjects with multiple-high-risk HPV infections. This may be related to sexual behaviour, social class (poverty and malnutrition), high parity, lack of barrier contraceptive protection and use of steroidal contraception, [7,12-16]. However, these confounders could not be assessed in our study because these data were not available. Furthermore, the risk of cervical cancer among women with multiple high-risk infections has not been well studied; therefore additional studies are warranted.

Studies show that younger age is associated with an increased risk of cervical HPV infection [17-20]. Therefore, for comparisons of HPV infections between different populations, age must be standardized. A limitation in our study is that although all subjects were recruited from the general population, the mean age for each country was different. Pittsburgh subjects were the oldest (mean age = 53 ± 9.0 years), and this was representative of the older population in the geographical region [21]. Jamaican women were the youngest population, also reflecting the demographics for the country [22]. To address this limitation, we have presented age-adjusted prevalence rates so that these values might be compared. Another limitation in this study is that only 11% (N = 10) of the US study population were African-American, which did not allow for adequate comparisons between African-American and Caribbean women. However, the proportion of African-American subjects is representative of the Pittsburgh demographics.

## Conclusion

The higher prevalence of cervical HPV infections and multiple high-risk infections in Caribbean and US-Black women may contribute to the high incidence and prevalence of cervical cancer in these populations. Further studies of the distribution of HPV types between ethnic groups and the risk of cervical cancer among women with multiple high-risk infections are currently ongoing.

## Competing interests

The authors declare that they have no competing interests.

## Authors' contributions

CR contributed to study design, sample/data collection for Tobago and US populations, performed the data analysis and wrote the manuscript. JBW performed the sample and data collection of the Tobago population. ET, contributed to the overall study design and the writing of the manuscript. AW, contributed to the study design, sample/data collection of the Jamaican population and performed the HPV testing. NM and RE: contributed to study design of the US population. AP, V W, CB and SMG: contributed to study design and sample/data collection for the Tobago population. SE: performed the HPV testing. HF, DG, MJ, MS, NY, NMA: contributed to the study design, sample/data collection of the Jamaican population. All co-authors reviewed and approved the manuscript.
